# Transforaminal Lumbar Interbody Fusion (TLIF) with Expandable Banana-Shaped Interbody Spacers—Institutional 5-Year Experience

**DOI:** 10.3390/jcm14155402

**Published:** 2025-07-31

**Authors:** Martin N. Stienen, Lorenzo Bertulli, Gregor Fischer, Linda Bättig, Yesim Yildiz, Laurin Feuerstein, Francis Kissling, Thomas Schöfl, Felix C. Stengel, Daniele Gianoli, Stefan Motov, Ethan Schonfeld, Anand Veeravagu, Benjamin Martens, Nader Hejrati

**Affiliations:** 1 Spine Center, Cantonal Hospital St. Gallen, HOCH Health Ostschweiz & University of St. Gallen, 9000 St. Gallen, Switzerland; martin.stienen@h-och.ch (M.N.S.); lorenzo.bertulli@h-och.ch (L.B.); gregor.fischer@h-och.ch (G.F.); linda.baettig@h-och.ch (L.B.); yesim.yildiz@h-och.ch (Y.Y.); laurin.feuerstein@h-och.ch (L.F.); francis.kissling@h-och.ch (F.K.); thomas.schoefl@h-och.ch (T.S.); felix.stengel@h-och.ch (F.C.S.); daniele.gianoli@h-och.ch (D.G.); stefan.motov@h-och.ch (S.M.); benjamin.martens@h-och.ch (B.M.); 2 Department of Neurosurgery, Cantonal Hospital St. Gallen, HOCH Health Ostschweiz & University of St. Gallen, 9000 St. Gallen, Switzerland; 3 Department of Orthopaedic Surgery, Cantonal Hospital St. Gallen, HOCH Health Ostschweiz & University of St. Gallen, 9000 St. Gallen, Switzerland; 4 Department of Neurosurgery, University of Stanford, Stanford, CA 94305, USA; ethan.schonfeld@stanford.edu (E.S.); anand.veeravagu@stanford.edu (A.V.); 5 Neurosurgery Artificial Intelligence Lab, Stanford University School of Medicine, Stanford, CA 94305, USA

**Keywords:** transforaminal lumbar interbody fusion, TLIF, expandable spacer, ALTERA, complications, sagittal parameters, outcome

## Abstract

**Background:** Transforaminal lumbar interbody fusion (TLIF) with static cages is a frequently performed procedure. Larger series focusing on the use of expandable TLIF spacers are less common. **Methods:** This retrospective, single-center observational cohort study reviewed consecutive patients treated by TLIF using expandable titanium interbody implants (ALTERA™, Globus Medical Inc., Audubon, PA, USA) for degenerative pathologies from L2-S1 between 11/2018 and 09/2023. Surgical parameters, adverse events, radiological outcomes (fusion rate, segmental lordosis, spinopelvic parameters), and clinical outcomes were analyzed through a mean postoperative follow-up of 12 months. **Results:** This study identified 270 patients (mean age 65 years, 50.4% female) who underwent TLIF with expandable interbody spacers at 324 levels. Clinical outcomes were good or excellent in 74.1% of patients at 3 months and 71.8% at 12 months. Radiographic fusion was achieved in 73.1% of assessable segments at 12 months. Segmental lordosis increased significantly from 17.8° preoperatively to 20.0° at 12 months (*p* < 0.001). Adverse event (AE) rates were acceptable across all timepoints, with no device failures or device-associated complications observed. **Conclusions:** This study demonstrates that TLIF with expandable titanium interbody implants was safe, associated with high fusion rates, and enabled significant restoration of segmental lordosis that was maintained during follow-up.

## 1. Introduction

Discogenic low-back pain arises from intervertebral disc degeneration involving multifactorial processes including mitochondrial dysfunction, advanced glycation end product accumulation, and inflammatory cascades [1]. When conservative management fails, surgical intervention with interbody fusion becomes necessary to restore spinal stability and alleviate symptoms.

Transforaminal lumbar interbody fusion (TLIF) is a frequently conducted surgical procedure, which allows for open or minimally invasive decompression and 360° fusion of a spinal motion segment via a single, transforaminal approach to the disc [2,3,4]. The use of an interbody cage helps to maintain or expand the intervertebral distance, allowing to increase foraminal height while helping with sagittal alignment restoration and increasing the chance of solid fusion [5]. Optimal spacer selection and placement may be challenging, however, as a certain implant height is required to obtain a fusion-inducing press fit according to Wolf’s law [6], while a too largely dimensioned implant can increase the risk of endplate injury and subsidence. The latter predisposes for instability, loss of lordosis, and suboptimal patient outcomes [7,8]. 

Expandable interbody spacers were developed to overcome these difficulties. They can be introduced into the disc space and positioned while at a contracted height, followed by controlled expansion until the desired device height and fit is obtained. In theory, the use of expandable cage technology may help to reduce the impaction force, endplate injury, and device subsidence [5]. Comparative analyses suggest that expandable cages may help to achieve a greater restoration of intervertebral distance (disc height) and segmental lordosis (SL), compared to static cages [5,9,10,11], however; this was not reproduced by other studies [12,13,14,15,16]. There have also been reports about cage failure with collapse, migration, and extrusion [17,18,19,20,21], highlighting the need for evaluating the safety of their use across a large sample.

The literature on expandable cage technology is limited, as mostly smaller case series and retrospective, single-center studies have been published so far [5,9,11,12,14,15,16,17,18,20,22,23,24,25,26,27,28,29,30]. The aim of this study was to complement the existing body of literature with our institutional experience, focusing on adverse events (AEs), clinical-radiological outcomes, and fusion rates in a large cohort.

## 2. Methods

### 2.1. Hospital Setting

The Cantonal Hospital of St. Gallen, Switzerland is an academic, tertiary teaching-hospital associated with the Medical School of St. Gallen. It serves a population of approximately 1,000,000 inhabitants. The Spine Center of Eastern Switzerland is formed mutually by twelve board-certified neurosurgeons or orthopedic spine surgeons and seven residents/physician assistants. Use and types of implants are unified. About 1100–1300 spine surgical procedures under general anesthesia are performed annually, including about 100–120 TLIF procedures annually. The ALTERA Expandable Integrated TLIF Spacer (Globus Medical Inc.) was introduced at our center in October 2018. This modern spacer is a banana-shaped, anteriorly placed, continuously expandable device made predominantly of titanium alloy with a central graft aperture. TLIF procedures were performed by all board-certified spine surgeons, or by fellows or senior residents under supervision.

### 2.2. Patient Identification

**Figure 1:**
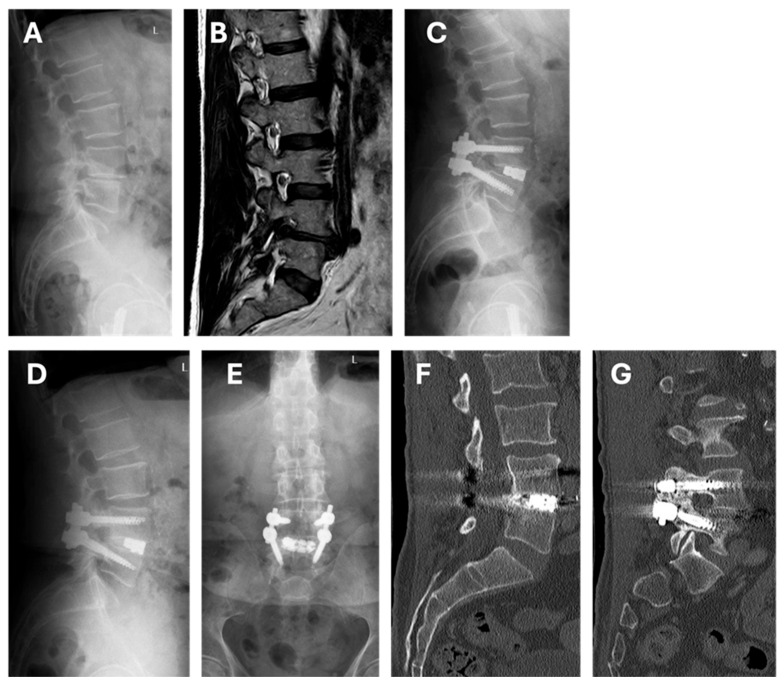
Case vignette of a 74-year-old male with right-sided radicular pain corresponding to both L4 and L5 dermatomes, as well as facet joint pain with short-term response to repeated injections. (**A**) Standing sagittal X-ray studies demonstrate degenerative grade I spondylolisthesis at L4/5 and collapsed disc height. PI 58° (Roussouly type 3), PT 23°, SS 35°, LL L1-S1 52°, SL at L4/5 of 18°. (**B**) Preoperative sagittal paramedian MRI on the right side, illustrating the foraminal stenosis due to disc protrusion and facet joint disease. A single-level TLIF was performed with unilateral facetectomy and implantation of a Globus Medical ALTERA spacer (9–13 mm expandable, 10 × 31 mm, 8° lordotic). (**C**) Standing X-ray imaging before discharge, demonstrating an increase in SL by 11° (to 29°). The patient made an unremarkable, excellent recovery with complete improvement of symptoms at 3 and 12 months. X-ray studies at 12 months in sagittal (**D**) and coronal (**E**) view. PT 12°, SS 46°, LL L1-S1 68°, SL at L4/5 29°. An abdominal CT scan, performed for an oncological workup about 2 years postoperatively showed evidence of solid intersomatic fusion ((**F**); classified as BSF-type 3) and unilateral solid fusion mass ((**G**); classified as Lenke-type B).

**Figure 2:**
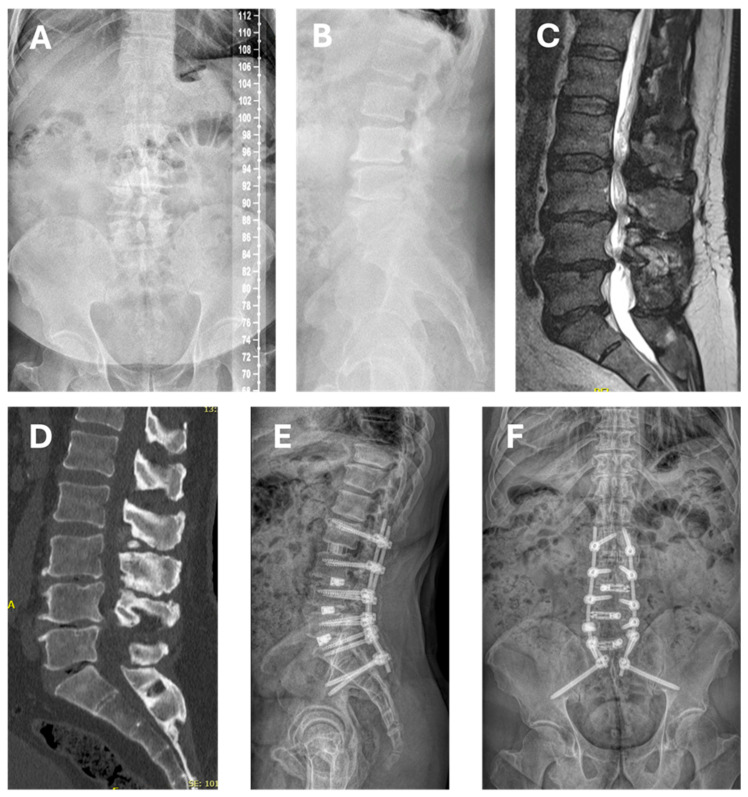
Case vignette of a 64-year-old male with history of three prior lumbar decompression procedures (hemilaminectomy L5, foraminotomy L5, bilateral decompression L3/4), presenting in our outpatient clinic with neurogenic claudication, recurring spinal stenosis, severe foraminal stenosis at multiple levels, and motor deficit for elevation of the left foot (BMRC grade 2/5). He received multiple epidural steroid injections and facet blocks with only temporary improvement of symptoms. (**A**,**B**) Standing preoperative whole-spine X-ray in anterior–posterior and lateral projection (PI 29°, Roussouly type 1, PT 5°, SS 24°, LL L1-S1 46° (ideal: 43° according to www.spinebit.io, accessed on: 27 July 2025), SL L4/S1 28° (ideal: 39° according to www.spinebit.io)). (**C**) Sagittal MRI demonstrating spinal stenosis at L2/3, L3/4, and L4/5. (**D**) Sagittal CT scan with evidence of degenerative disc disease and vacuum sign at L3/4, L4/5, and L5/S1. Considering the severity of his symptoms (VAS back pain 7/10, VAS leg pain 10/10, Oswestry Disability Index (ODI) 53.3/100%, Core Outcome Measures Index (COMI) Back 8.14/10), a fusion procedure was proposed entailing indirect decompression at L2/3 via extreme lateral lumbar interbody fusion (XLIF), as well as direct decompression with TLIF at L3-S1. (**E**,**F**) Postoperative standing whole-spine X-ray in anterior–posterior and lateral projection demonstrates good restoration of SL between L4-S1 (37°) and between L1-S1 (42°). The patient recovered well, without suffering from intra- or postoperative complications. At 12 months, he presented with significant improvement of preoperative symptoms (VAS back pain 3/10, VAS leg pain 1/10, ODI 22/100%, COMI Back 2.28/10) and without signs of pseudarthrosis.

Unique patient numbers (UPNs) of patients, where an ALTERA spacer was employed until September 2023 were identified by electronic review of our hospital’s purchasing department. In addition, the surgery schedules were cross-checked for any TLIF procedures to make sure those with an expandable implant were included. Consecutive adult patients with degenerative pathology who underwent single- (Figure 1) or multi-level (Figure 2) lumbosacral (L2-S1) TLIF were included. Cases were excluded where static TLIF cages were employed or where expandable TLIF spacers had been inserted outside the approved range of CE marked indications (e.g., use in the thoracic spine, for trauma, tumors, or infection).

### 2.3. Data Collection and Variables

Eleven surgeons retrospectively reviewed the electronic patient charts to extract relevant information, based on a pre-specified codebook to classify and define variables. Baseline information, including age, sex, body mass index (BMI; in kg/m^2^) [31], smoking status [32,33], American Society of Anesthesiology (ASA) grading scale of surgical risk, Charlson Comorbidity Index [34] (CCI; stratified into very low (CCI 0), mild (CCI 1&2), moderate (CCI 3&4) and severe (CCI > 4) according to Keller et al. [35], and the Canadian clinical frailty scale [36] (ranging from 1 (very fit) to 9 (terminally ill)), was collected prospectively before each surgical procedure. Moreover, the indication for surgery was determined.

Surgical parameters included the spinal level where the TLIF was performed (between L2-S1), the total number of operated segments (e.g., 1 segment for L4/5; 2 segments for L3/L5), the type of expandable interbody spacer (8° or 15° lordotic), additional use of other types of interbody fusion procedures during the same anesthesia (e.g., anterior lumbar interbody fusion (ALIF), (extreme) lateral lumbar interbody fusion (XLIF/LLIF), posterior lumbar interbody fusion (PLIF), other), type of laminectomy (partial/complete), length of surgery (in minutes), estimated blood loss (EBL; in ml; defined by anesthesiologists from suction drainage and swabs), and intraoperative AEs.

Postoperative AEs at time of discharge from hospital were considered as documented within the discharge summary and the electronic patient record. Severity of AEs was classified according to the Therapy-Disability-Neurology (TDN) scoring system [37]. The same was recorded at 3 months (3M; AEs occurring between discharge and 3M follow-up) and at 12 months (12M; AEs occurring between 3M and 12M follow-up). Clinical outcome was graded according to Macnab into four categories, as best estimated from the follow-up letter and the electronic patient chart (excellent, good, fair, poor) [38,39,40]. To assess the degree and rate of fusion, intersomatic fusion was evaluated according to the Brantigan, Steffee & Fraser (BSF) classification [41], which distinguishes between obvious pseudarthrosis, intermediate type, and solid fusion (Figure 1). To evaluate the posterolateral fusion mass, the classification by Lenke et al. [42] was applied, which differentiated between definitely solid, possibly solid, probably not solid, and definitely not solid (Figure 1). A segment was considered radiographically fused if graded as either a solid fusion of the interbody space or a Lenke-type A or B grade of the posterolateral masses, or both. The radiographic fusion rate was then calculated based on the number of assessable segments, which were those with readable images for either intersomatic or posterolateral fusion sites, or both.

As computed tomography (CT) imaging was not available for all patients and ratings performed on (scoliosis) X-ray imaging were less certain, a “clinical pseudarthrosis” category was added, where the diagnosis of pseudarthrosis in patients without any pain was considered unlikely, but in patients with the typical delayed onset of recurring axial or radicular pain weeks to months after the index operation, pseudarthrosis as a failed attempt of spinal fusion was considered likely [43]. Diagnosis of “clinical pseudarthrosis” was based on both the clinical presentation in conjunction with available imaging studies (X-ray, CT, and Single Photon Emission Computed Tomography (SPECT)), after ruling out other causes of persistent pain.

The baseline sagittal Cobb angle (SL; e.g., upper endplate of L4 vertebra to lower endplate of L5 vertebra for the L4-L5 segment), as well as spinopelvic parameters including pelvic incidence (PI), lumbar lordosis (LL; from L1-S1), pelvic tilt (PT), sacral slope (SS), and C7-sagittal vertebral axis (C7 SVA), were measured using standing (scoliosis) or EOS X-ray images, whenever available. The patient-specific “ideal LL” was calculated using a web-based app (http://www.spinebit.io), which is based on the formulas by Le Huec and the European Spine Study Group [44,45,46]. Based on these values, the PI-LL mismatch (PI minus LL) and the ideal–actual LL mismatch (“ideal LL” minus actual LL) were determined. Postoperative radiological outcomes at 3M and 12M again included SL, LL, PT, SS, and C7 SVA. The intervertebral distance in mm was measured to determine the height of the disc space at its anterior, middle, and posterior aspect before and after surgery.

### 2.4. Surgical Technique

Pedicle screws were placed first to perform reduction maneuvers and to allow for some initial posterior distraction after uni- or bilateral partial facetectomies. Then, depending on the pathology and symptoms, either a uni- or bilateral complete facetectomy was performed to decompress the exiting nerve root and prepare the Kambin triangle(s) as entry zone(s) for the uni- or biportal TLIF procedure. The choice between the uniportal and biportal TLIF approach was determined based on several factors, including the extent of decompression required, need for bilateral cage placement, and surgeon preference. The uniportal approach was typically selected for single-level procedures with adequate unilateral access, while the biportal approach was employed when bilateral decompression or extensive coronal correction was required. After superficial discectomy, an intersomatic “Chiari” spreader was introduced for final segmental distraction, followed by a deep discectomy until the ALL was visualized. The ALL was preserved, in all patients. The TLIF cage was chosen in terms of width and height after trialing, and the desired degree of lordosis (8° or 15°) was chosen based on anatomical and physiological considerations. Following application of autogenous and/or allogeneic bone graft into the anterior aspect of the disc space, the TLIF cage was inserted, positioned, and expanded in its final position under serial fluoroscopic control. After releasing the introducer, the remaining disc space was filled with bone graft and posterior compression was applied as needed to increase SL.

### 2.5. Statistical Analysis

Statistical Analysis Software (SAS) (version 9.4; SAS Institute Inc., Cary, NC, USA) was used and employed mostly for descriptive statistics, reporting results as mean (standard deviation; SD) or count (percent). For the analysis of sagittal spinal parameters over time, outcomes were compared to the preoperative value using paired *t*-tests with *p* < 0.05 considered statistically significant.

### 2.6. Ethical Considerations

The Institutional Review Board (IRB) of Eastern Switzerland approved the study (BASEC ID 2023-01343). Retrospective collection, analysis, and publication of anonymized patient data were allowed with an institutional waiver for informed consent.

## 3. Results

### 3.1. Patient Cohort

**Figure 3:**
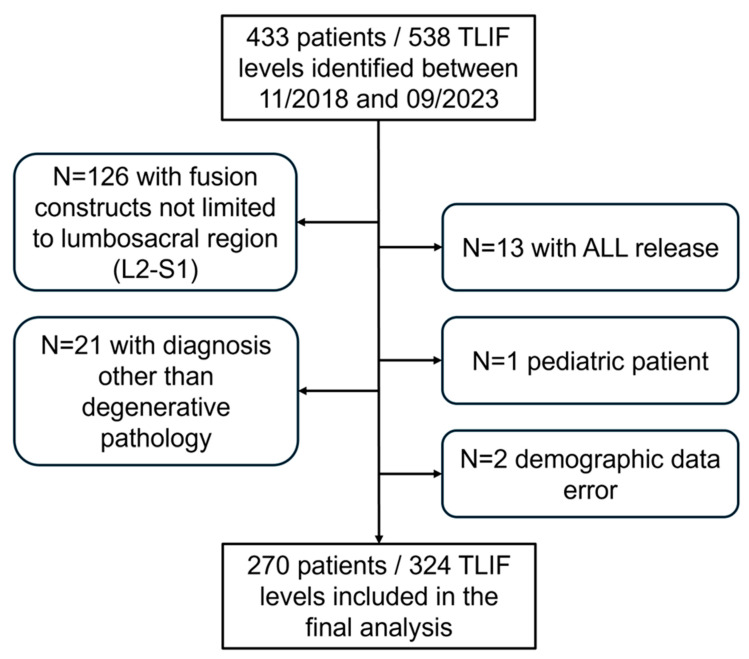
Flowchart of inclusion and exclusion of patients undergoing TLIF with use of an expandable ALTERA spacer between November 2018 and September 2023 in our institution.

This study identified a total cohort of 433 patients who underwent a TLIF procedure using an expandable ALTERA cage at 538 levels. After considering all predefined inclusion and exclusion criteria, 270 patients with TLIF performed at 324 levels remained in the analysis (Figure 3).

The mean age of the cohort was 64.6 years (SD 12.4); 50.4% of patients were female. A total of 66.3% of patients were overweight (BMI ≥ 25–30 kg/m^2^) or obese (BMI > 30 kg/m^2^), 41.9% were smokers or had a previous history of smoking, and approximately one out of four patients had moderate or severe comorbidities. All procedures were performed for a degenerative pathology, including spondylolisthesis, spinal stenosis with suspected instability, or (recurrent) disc herniation. More detailed baseline demographic information can be found in Table 1.

Table 2 summarizes procedure-related parameters. A total of 83% of TLIFs were performed between L4-S1, frequently as mono- or bisegmental fusion procedures or as part of longer constructs, including 3–4 segments of fusion; 13% of procedures entailed additional modalities of interbody fusion such as an ALIF, XLIF/LLIF, PLIF, or other approach. The average number of operated segments was 1.5 per patient (SD 0.7). The duration of surgery was about 4.5 h on average and the mean EBL was 556 mL (SD 446). Intraoperative AEs were encountered in 31 procedures (11.5%), of which the majority were incidental durotomies (74.2%).

### 3.2. Follow-Up and Reasons for Missing Data

Patients were discharged after a mean length of stay of 9.4 (SD 6.6) days. The 3-month follow-up was completed by *n* = 257 (95.2%) patients at a mean of 87.5 (SD 44.1) days postoperative. The 12-month follow-up was completed by *n* = 210 (77.8%) patients at a mean of 361.6 (SD 111.5) days postoperative. The main reasons for dropping out during the last follow-up included (i) surgeries, which were performed within less than 12 months, (ii) patients seeking further care at another facility, or iii) patients moving away from the area.

### 3.3. Clinical Outcomes

Between surgery and hospital discharge, AEs were noted in *n* = 53 patients (19.6%), of which most were anemia (24.5%), wound healing disorders (15.1%), or new neurological deficits/neuropathic pain (11.3%). Five (9.4%) complications were severe, and none led to death. Medical complications (67.9%) were more common than surgery-related complications (33.1%; Table 3).

At 3 months, AEs were noted in *n* = 27 patients (10.0%), of which most were new fractures/hardware-related issues (48.1%), wound healing disorders (33.3%), or adjacent segment disease (ASD; 14.8%). Most AEs were considered surgery-related (96.3%). Two AEs were graded as severe (7.4%), and there was no mortality at this time point. The clinical outcome was excellent or good in 74.1% (189/255) of patients with documented outcomes. Non-union was suspected in 7.5% of assessable TLIF segments.

At 12 months, AEs were noted in *n* = 25 patients (9.3%), of which most were ASD (52.0%), pseudarthrosis (24.0%), or new fractures/hardware-related issues (12.0%). Of AEs reported, one AE was graded severe (4%) and one related to discitis and sepsis was graded deadly (4.0%). The clinical outcome was excellent or good in 71.8% (148/206) of patients with documented outcomes. Radiographic fusion was observed in 73.1% of assessable TLIF segments, and clinical non-union was suspected in 12.0% of segments. No instance of cage collapse was observed through final follow-up.

### 3.4. Radiological Outcomes

Table 4 illustrates the baseline spinopelvic measurements, as well as the evolution of the sagittal radiological parameters over time. There was a significant postoperative increase in both the global LL and SL, which were preserved over time at 12 months postoperatively, leading to significant decreases in both the PI-LL mismatch and ideal–actual LL mismatch. The intervertebral distance significantly increased by 4.5 mm in the anterior, 3.6 mm in the middle, and 1.9 mm in the posterior aspect of the disc space (all *p* < 0.001). This distance slightly decreased at the time of 3- and 12-month follow-up but remained significantly greater than prior to surgery.

**Figure 4:**
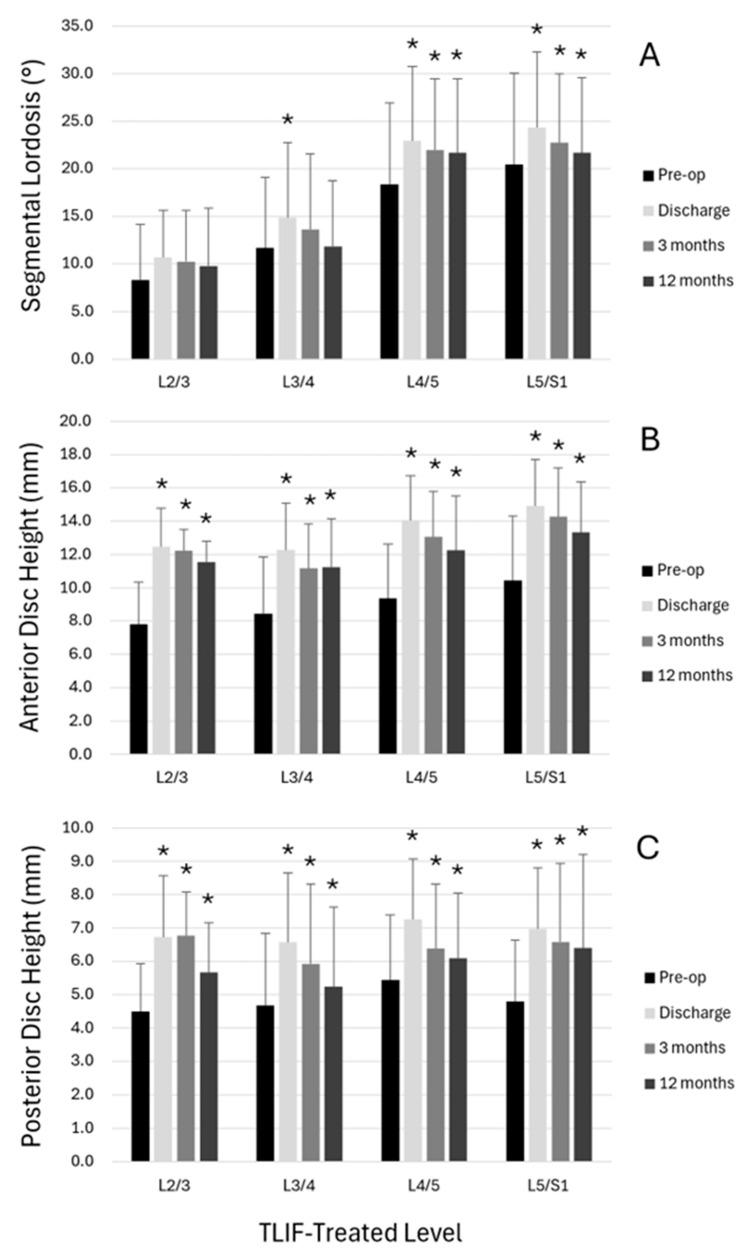
Radiographic outcomes by spinal level showing (**A**) segmental lordosis with significant sustained improvements at L4/5 and L5/S1 levels, (**B**) anterior disc space height with significant increases maintained at all levels, and (**C**) posterior disc space height demonstrating sustained improvements across all treated levels. (* *p* < 0.05 vs. preoperative values).

Radiographic parameters specific to TLIF-treated segments were also evaluated by spinal level (Figure 4). Preoperatively, SL and disc space heights were observed to vary by level of treatment. SL was significantly increased at discharge from preoperative values at L3/4, L4/5, and L5/S1, and significant increases were sustained at 3 months and 12 months at L4/5 and L5/S1 (*p* < 0.05). Significant increases were observed in anterior and posterior disc space heights at all spinal levels and all postoperative times compared to preoperative measurements (*p* < 0.05).

## 4. Discussion

Expandable cage technology is relatively novel, and while numerous studies have explored the clinical impact and radiographic performance, the choice of interbody technology continues to incite development of new devices and considerable interest by clinicians. These factors have prompted the current analysis of an institutional 5-year experience consisting of 270 patients and 324 segments implanted with a specific anterior TLIF expandable spacer (ALTERA). The present study results may contribute to the decision-making pathway of patient selection and provide insight into contextualizing improvement when using expandable, banana-shaped spacers in TLIF procedures.

Notably, our spine center serves as a reference center for surrounding smaller and private hospitals and is responsible for taking care of patients that are rejected for surgery elsewhere, or that have suffered from complications at outside hospitals before. Despite our cohort including patients with considerable comorbidities associated with adverse outcomes, such as considerable degrees of frailty, poor bone quality, smoking, and obesity [31,32,33], our study reports detailed adverse events rates, including intraoperative (11.5%), at time of discharge (19.6%), and during 3- and 12-months of follow-up (~9–10%). Importantly, collapsed or loosened TLIF implants that migrated into the spinal canal or foramen, or that resulted in any kind of (neural) injury, as was previously reported for alternative expandable TLIF devices, were not identified [17,19,20,21,47]. Thus, the investigated ALTERA expandable TLIF device functioned reliably in our study series with respect to maintenance of the expanded device height.

Achieving appropriate spinopelvic alignment through restoration or preservation was considered a central goal of surgery for the present patient cohort. Even in short-construct lumbar fusion procedures for degenerative conditions, Leveque et al. have recommended that spinopelvic alignment be assessed and confirmed to be normal (PI-LL < 10°) before, during, and after surgery to enhance the likelihood of successful outcomes and reduce the risk of adjacent segment disease [48]. In the present study of degenerative (non-deformity) pathologies, the preoperative spinopelvic alignment measure of PI-LL averaged 5.5° and was within the normal range [47] for the majority of patients, indicating that in addition to symptomatic improvement, alignment preservation was a surgical goal for the majority of patients. Following treatment with fusion constructs including one or more expandable TLIF interbody cages, average PI-LL mismatch significantly improved by 2.2° by the final 12-month timepoint.

In addition to regional spinopelvic alignment changes, segment-specific changes were also observed. At TLIF-treated segments specifically, the use of expandable interbody cages allowed for a restoration of 4.0° of SL at the time of discharge, and 2.1° at 12-month follow-up. Our results compare well to Ledesma et al., who achieved 2.8° of additional SL at 6 weeks and 2.5° at 12 months in their cohort of 1- or 2-level MIS TLIF patients treated with an expandable interbody spacer [5]. Similarly, Chen et al. reported a gain of 1.4° and 2.0° immediately and at last follow-up in *n* = 30 patients treated by single-level TLIF with use of an expandable cage [24]. Our results demonstrate the inherent design of the anterior convexity angle of the banana-shaped expandable spacer and the anterior placement within the disc space, as anterior intervertebral distance significantly increased by an average of 2.9 mm at final follow up, thus supporting the recovery and preservation of SL correction [49]. While height changes were most pronounced in the anterior aspect of the disc space where cages were placed, posterior disc space height was also observed to increase significantly from the preoperative period to each postoperative period, potentially supplementing any direct decompression of posterior neural structures achieved via the TLIF and laminectomy procedures.

Our cohort captures the initial experience and technique evolution of using the banana-shaped expandable spacer over a 5-year period. Based on our experience on the use of expandable cages, surgeons may be tempted to spend less time on the discectomy and endplate preparation. It seems possible to insert the cage early and expand it before a thorough segmental release has been achieved, which may lead to endplate injury and cage subsidence. Observations of cage subsidence were most pronounced early in this case series, and these along with settling and bony remodeling may partially account for some of the postoperative trends seen in the spinopelvic alignment measures, notably the decreasing SL and disc space heights compared to discharge. Future research efforts focused on evaluating subsidence using a standardized grading scheme with correlation to patient characteristics and clinical outcomes may be desirable for various expandable cage technologies.

Overall, a successful outcome (rated as excellent or good) according to the Macnab criteria [39] was achieved by 74.1% of the patients with recorded outcomes at 3 months and 71.8% at 12 months, with 90.6% and 93.2% of patients demonstrating symptomatic improvement (rated from excellent to fair). Radiographic fusion was achieved in 73.1% of assessable segments at 12 months, while the interbody fusion rate was lower at 44.0%, with another 41.7% being BSF intermediate type. The interbody fusion outcome is similar to a rate (45.5%) recently reported by Liu et al. following a rigorous radiographic review of single-level TLIFs without rhBMP-2 using a modified BSF classification with minimum 2-year follow-up [50]. Lastly, the clinical non-union rates among assessable segments were 7.5% at 3-months and 12.0% at 12-months follow-up (Table 3), which is similar to those of prior studies reporting 6.7–6.9% after 1- or 2-level MIS TLIF [9,29], 7% at 12 months after 1- or 2-level MIS-TLIF [18,20], or 9.8% at 12 months after MIS-TLIF [28].

### 4.1. Implications for Practice

Based on our experience with the use of the ALTERA expandable TLIF spacer technology, it has been observed to be a well-working implant that functions reliably to gain disc height and SL, and achieve surgical goals. No collapsed implants were detected by 12 months, and no other AEs that were directly related to implant malfunction were recorded. While static TLIF cages may represent viable alternatives for “standard cases”, expandable cages, in our opinion, have certain advantages, especially in the settings of MIS approaches (possibility to insert a smaller implant and expand it in situ) and to assist with controlled correction of coronal and/or sagittal spinal malalignment by placing and expanding the cages in the desired location of the intervertebral space. While our study included both open and minimally invasive approaches, expandable cage technology may offer particular advantages in MIS-TLIF procedures by allowing insertion of a smaller-profile device that can subsequently be expanded in situ, potentially reducing tissue trauma and facilitating recovery. However, our study design did not specifically compare MIS versus open approaches, and future research should evaluate whether the theoretical advantages of expandable cages are more pronounced in minimally invasive settings.

### 4.2. Strengths and Weaknesses

Our study reports data from a large cohort of consecutive patients, treated with expandable TLIF spacers. This is an innovative technology, with increasing popularity, but on which there are limited data [5,9,10,11,12,14,15,16,17,18,20,22,23,24,25,26,27,28,29,30,51,52,53,54]. Despite its retrospective character, the missing data burden was reasonably low, especially for intraoperative and perioperative safety outcomes. Our series includes a very detailed record of perioperative AEs and grading of their severity using the TDN scale [37], which distinguishes it from previous studies.

Selection bias is likely to be present, as there was no control for the reason why patients were receiving an expandable TLIF spacer versus a static TLIF cage or any other type of anterior or posterior interbody fusion. The standardized use of patient-reported outcome measures (PROMs) in our center was only introduced in 2022, which is why this study classified the functional outcome using the Macnab criteria with a simple 4-tier scale [39,40,55]. A further limitation of this study is the absence of CT scan images for the assessment of the fusion status in all patients, as those are not routinely performed as part of the follow-up regimen in patients faring well. However, if patients developed clinically relevant symptoms on follow-up, CT or SPECT scans were ordered to rule out pseudarthrosis. Finally, there was no control group, as it was not the aim of the study to conduct a comparative analysis. In our series, 13% of patients (*n* ≈ 35) underwent additional interbody fusion procedures during the same anesthesia. While this represents a relatively small subset, it may have influenced overall outcomes, though the small numbers preclude meaningful subgroup analysis.

## 5. Conclusions

Data collected in this study suggest that the use of TLIF with an anteriorly placed expandable titanium interbody implant in this series promoted radiographic fusion, enabled correction and preservation of spinopelvic alignment, and was associated with an acceptable adverse event profile through 12 months of follow-up.

## Figures and Tables

**Table 1:** Demographic information of *n* = 270 patients undergoing thoracolumbar fusion surgery including the use of an expandable interbody spacer on 324 levels. Data are presented as count (percent) or mean (standard deviation; SD). ASA = American Society of Anesthesiology; BMI = body mass index; CCI = Charlson comorbidity index; CFI = Canadian frailty index.

Variable	
Age (years)	64.6 (SD 12.4)
*Sex*	
Female	136 (50.4%)
Male	134 (49.6%)
*BMI categories, according to the WHO*	
Underweight (<18.5 kg/m^2^)	2 (0.7%)
Healthy (18.5–24.9 kg/m^2^)	89 (33.0%)
Overweight (25–30 kg/m^2^)	100 (37.0%)
Obese (>30 kg/m^2^)	79 (29.3%)
*Smoking status*	
Nonsmoker	157 (58.1%)
Smoker	85 (31.5%)
Former smoker	28 (10.4%)
*ASA grade*	
I	7 (2.6%)
II	169 (62.6%)
III	90 (33.3%)
IV	4 (1.5%)
*CCI severity*	
Very low	113 (41.9%)
Mild	103 (38.1%)
Moderate	37 (13.7%)
Severe	17 (6.3%)
*CFI severity*	
Very fit	22 (8.1%)
Well	82 (30.4%)
Managing well	97 (35.9%)
Vulnerable	57 (21.1%)
Mildly frail	8 (3.0%)
Moderately frail	3 (1.1%)
Severely frail	1 (0.4%)
*Degenerative disease type*	
(Recurrent) disc herniation	42 (15.6%)
Spinal stenosis with instability	62 (23.0%)
Degenerative spondylolisthesis	75 (27.8%)
Isthmic spondylolisthesis	38 (14.1%)
Other	53 (19.6%)
**Total**	***n* = 270 (100%)**

**Table 2:** Surgery-specific information of *n* = 270 patients undergoing lumbar fusion surgery including the use of an expandable interbody spacer in *n* = 324 segments. Data are presented as count (percent) or mean (standard deviation, range) of patients (*), segments (**), or adverse events (***). AE = adverse event; ALIF = anterior lumbar interbody fusion; L = lumbar; LLIF = lateral lumbar interbody fusion; PLIF = posterior lumbar interbody fusion; S = sacral; TLIF = transforaminal lumbar interbody fusion; XLIF = extreme lateral lumbar interbody fusion.

Variable	
TLIF segment **	
L2/3	11 (3.4%)
L3/4	44 (13.6%)
L4/5	164 (50.6%)
L5/S1	105 (32.4%)
Number of operated segments *	1.5 (SD 0.7, range: 1–4)
Type of interbody spacer **	
8° lordotic	161 (49.7%)
15° lordotic	163 (50.3%)
Other types of interbody fusion employed *	
None	234 (86.7%)
XLIF/LLIF	7 (2.6%)
ALIF	14 (5.2%)
PLIF	12 (4.4%)
Other	3 (1.1%)
Type of laminectomy **	
Partial	231 (71.3%)
Complete	93 (28.7%)
Length of surgery, in minutes *	263 (SD 89, range: 124–725)
Estimated blood loss, in milliliters *	556 (SD 446, range: 50–3600)
Intraoperative AE *	
No	239 (88.5%)
Yes	31 (11.5%)
Type of intraoperative AE ***	
Dural lesion	23 (74.2%)
Hardware-related	5 (16.1%)
Osseous injury	2 (6.5%)
Excessive bleeding	1 (3.2%)

**Table 3:** Information on surgical adverse events (AEs) and outcome at discharge, 90 days and 12 months in *n* = 270 patients undergoing lumbar fusion surgery including the use of an expandable interbody spacer in *n* = 324 segments. Data are presented as count (percent) or mean (standard deviation). n/a = not applicable; TDN = Therapy-Disability-Neurology.

Variable	Discharge	90 Days	12 Months
*AE*			
No	217 (80.4%)	227 (84.1%)	185 (68.5%)
Yes	53 (19.6%)	27 (10.0%)	25 (9.3%)
Missing data	0 (0%)	16 (5.9%)	60 (22.2%)
*Type of AE ^#^*			
Medical	36 (67.9%)	1 (3.7%)	0 (0%)
Surgical	17 (33.1%)	26 (96.3%)	25 (100%)
*TDN grade of AE*			
1 (mild AE)	7 (13.2%)	1 (3.7%)	0 (0%)
2 (mild to moderate AE)	22 (41.5%)	2 (7.4%)	5 (20.0%)
3 (moderate AE)	19 (35.8%)	22 (81.5%)	18 (72.0%)
4 (severe AE)	5 (9.4%)	2 (7.4%)	1 (4.0%)
5 (death)	0 (0%)	0 (0%)	1 (4.0%)
*Clinical outcome*	n/a		
Excellent		92 (34.1%)	93 (34.5%)
Good		97 (35.9%)	55 (20.4%)
Fair		42 (15.6%)	44 (16.3%)
Poor		24 (8.9)	14 (5.2%)
Missing data		15 (5.6%)	64 (23.7%)
*Posterolateral fusion*	n/a	Assessable levels *n* = 220	Assessable levels *n* = 209
Definitively not solid		11 (5.0%)	17 (8.1%)
Probably not solid		138 (62.7%)	51 (24.4%)
Possibly solid		55 (25.0%)	83 (39.7%)
Definitively solid		16 (7.3%)	58 (27.8%)
*Intersomatic fusion*	n/a	Assessable levels *n* = 221	Assessable levels *n* = 216
Fusion		10 (4.5%)	95 (44.0%)
Intermediate type		183 (82.8%)	90 (41.7%)
Pseudarthrosis		28 (12.7%)	31 (14.4%)
*Any radiographic fusion*	n/a	Assessable levels *n* = 222	Assessable levels *n* = 216
Fused		71 (32.0%)	158 (73.1%)
Not fused		151 (68.0%)	58 (26.9%)
*Clinical non-union*	n/a	Assessable levels *n* = 279	Assessable levels *n* = 249
No		258 (92.5%)	219 (88.0%)
Yes		21 (7.5%)	30 (12.0%)
*Cage collapse*	n/a	n/a	Assessable levels *n* = 250
No			250 (100%)
Yes			0 (0.0%)

^#^ Type of AEs at discharge: *n* = 13 anemia (24.5%), *n* = 8 wound healing disorder/infection/hematoma (15.1%), *n* = 6 new neurological deficit/neuropathic pain (11.3%), *n* = 5 other AEs (9.4%), *n* = 4 pulmonary embolism (7.5%), *n* = 3 congestive heart failure (5.7%), *n* = 3 urinary tract infection (1.4%), *n* = 3 fracture/hardware-related issues (5.7%), *n* = 3 delirium (5.7%), *n* = 3 renal failure (0.9%), *n* = 2 pneumonia (3.8%). Type of AEs at 90 days: *n* = 13 fracture/hardware-related issues (48.1%), *n* = 9 wound healing disorder/infection/hematoma (33.3%), *n* = 4 adjacent segment disease/proximal junctional kyphosis or failure (14.8%), *n* = 1 pneumonia (3.7%). Type of AEs at 12 months: *n* = 13 adjacent segment disease/proximal junctional kyphosis or failure (52%), *n* = 6 pseudarthrosis (24%), *n* = 3 fracture/hardware-related issues (12%), *n* = 3 wound healing disorder/infection/hematoma (12%).

**Table 4:** Information on spinopelvic parameters and intervertebral distance in *n* = 270 patients undergoing thoracolumbar fusion surgery including the use of an expandable interbody spacer in 324 segments. Data are presented as count (percent) or mean (standard deviation), comparing measurements at follow-up with preoperative measurements. ° = degrees, LL = lumbar lordosis, C7 SVA = C7 sagittal vertical axis, PI = pelvic incidence, PT = pelvic tilt, SS = sacral slope.

Spinopelvic Parameters	Preoperative	Discharge		90 Days Postoperative		12 Months Postoperative	
**PI, in °**	**57.1 (12.1)**						
**Total LL, in °**	**51.6 (14.2)**	**49.8 (12.7)**	0.003	53.6 (12.5)	0.025	53.8 (12.3)	0.004
**SS, in °**	**38.0 (9.7)**	**37.0 (8.8)**	0.030	38.5 (9.0)	0.816	39.1 (8.6)	0.030
**PT, in °**	**19.0 (8.4)**	**19.9 (8.9)**	0.037	18.8 (8.6)	0.850	18.1 (9.1)	0.072
**Segmental lordosis, in °**	**17.8 (9.3)**	**21.9 (8.6)**	<0.001	20.8 (8.2)	<0.001	20.0 (8.6)	<0.001
**C7 SVA, in cm**	**4.2 (3.9)**	**4.1 (3.3)**	0.173	5.0 (3.5)	0.468	5.8 (4.6)	0.514
Ideal LL, in °	58.0 (7.4)						
Ideal-actual LL mismatch, in °	6.4 (12.2)	8.2 (11.1)	0.003	4.5 (11.2)	0.025	4.0 (11.4)	0.004
PI-LL mismatch, in °	5.5 (11.9)	7.2 (11.8)	0.003	3.7 (11.7)	0.032	3.3 (12.4)	0.004
Intervertebral distance, in mm							
Anterior disc space	9.6 (3.5)	14.0 (2.9)	<0.001	13.2 (2.9)	<0.001	12.5 (3.2)	<0.001
Middle disc space	8.2 (2.8)	11.8 (2.3)	<0.001	11.0 (2.2)	<0.001	10.5 (2.4)	<0.001
Posterior disc space	5.1 (1.9)	7.1 (1.9)	<0.001	6.4 (2.1)	<0.001	6.1 (2.3)	<0.001

## Data Availability

Dataset available on request from the authors.
